# Punctate inner choroidopathy with atypical presentation

**DOI:** 10.5935/0004-2749.20210018

**Published:** 2025-02-02

**Authors:** Juliana Albano, Maria Campos Pires, Marcelo Paccola

**Affiliations:** 1 Department of Ophthalmology, Faculty of Medical Sciences, Universidade Estadual de Campinas, Campinas, SP, Brazil; 2 School of Medicine, Universidade de Itaúna, Itaúna, MG, Brazil

**Keywords:** Choroiditis, Multimodal imaging, Tomography, optical coherence, Optical imaging, Fluorescein angiography, Coroidite, Imagem multimodal, Tomografia de coerência óptica, Imagem óptica, Angiofluoresceinografia

## Abstract

We report a case of a young Caucasian female presenting with sudden decrease of vision in the left eye, metamorphopsia, and nasal scotoma. Past medical history revealed a diagnosis of myasthenia gravis, which was currently treated with azathioprine, pyridostigmine, and prednisone. Ophthalmological examination showed fundus with clear vitreous and yellow-white lesions that were isolated and perimacular in the right eye, multiple and confluent in the macula, and punctate in periphery in the left eye. Laboratory workup ruled out the presence of infectious and inflammatory diseases. Fundus autofluorescence disclosed hypoautoflurescence with hyperfluorescent margins corresponding to the lesions observed in both eyes and the angiogram revealed hyperfluorescence since early phases without late leakage. Spectral-domain optical coherence tomography showed areas of intermittent retinal pigment epithelium elevations and disruption of the ellipsoid zone. She was diagnosed with punctate inner choroidopathy and then treated with an increased dose of daily prednisone, which resulted in progressive improvement of her visual acuity and anatomical status.

## Abstract (pt)

Relato de caso de mulher jovem, caucasiana, com súbita diminuição de acuidade visual de olho esquerdo, metamorfopsia e escotoma nasal. Apresentava diagnóstico de Miastenia gravis, em tratamento com Azatioprina, Piridostigmina e Prednisona. Fundo de olho demonstrava vítreo límpido e lesões amarelo-esbranquiçadas, perimaculares e isoladas em olho direito, múltiplas e confluentes em mácula e pontilhadas em periferia no olho esquerdo. Exames laboratoriais descartaram doenças infecciosas e inflamatórias. Auto-fluorescência revelou lesões hipoautofluorescentes com margens hiperfluorescentes correspondentes às observadas em ambos os olhos, enquanto angiofluoresceinografia mostrou hiperfluorescência desde as fases iniciais sem vazamento tardio. Tomografia de coerência óptica de domínio espectral revelou áreas de elevações intermitentes do epitélio pigmentar da retina e interrupção da zona elipsóide correspondente. Definiu-se como diagnóstico a coroidopatia interna ponteada, sendo instituído aumento na dose diária de Prednisona, com melhoria progressiva da acuidade visual e do aspecto de fundo de olho da paciente.

## INTRODUCTION

The white dot syndromes (WDS) are a group of inflammatory disorders that affect the outer retinal layers, the retinal pigment epithelium (RPE), and the choroid and include birdshot chorioretinopathy, multiple evanescent white dot syndrome, acute posterior multifocal placoid pigment epitheliopathy (APMPPE), multifocal choroiditis with panuveitis (MCP), serpiginous choroiditis, punctate inner choroidopathy/ multifocal choroiditis (PIC/MFC), and relentless placoid chorioretinitis^(1)^. Punctate inner choroidopathy (PIC) is an inflammatory syndrome that primarily affects myopic women in their second or third decade of life. It is characterized by multifocal, small, yellow-white spots at the level of deep retina and choroid in the posterior pole that evolve into atrophic chorioretinal scars without signs of anterior uveitis or vitritis^(2)^. Despite the progress made in differentiating each of the WDS, an overlap can occur and a definite diagnosis is not always possible. Recently, a classification system based on multimodal imaging was proposed by Raven and colleagues to differentiate the WDS through the findings on fundus autofluorescence (FAF), fluorescein angiography (FA), indocyanine green angiography (ICG), and spectral-domain optical coherence tomography (SD-OCT)^(3)^. Herein, we report a case of an unusual presentation of PIC in a young female with a previous diagnosis of myasthenia gravis and concomitant use of azathioprine, prednisone, and pyridostigmine. Fundus aspect resembled a placoid disease; however, multimodal imaging demonstrated features typically found in PIC, configuring this as a diagnostic challenge.

## CASE REPORT

**Figure 1:**
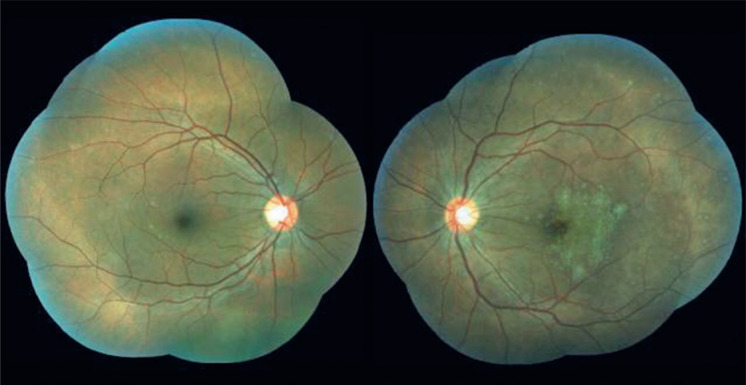
Fundus imaging of right and left eyes.

**Figure 2:**
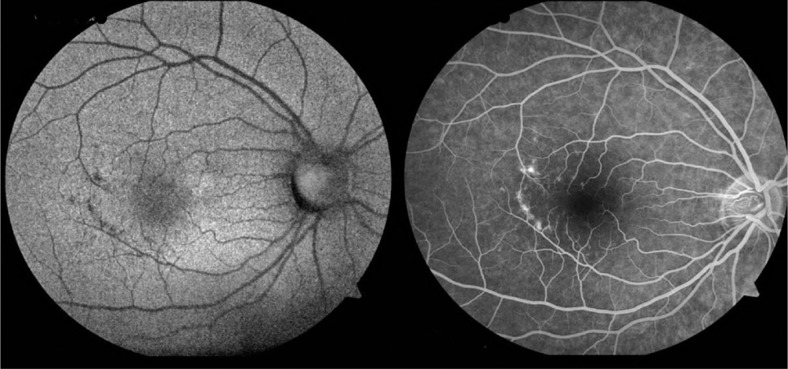
Autofluorescence (left) and fluorescein angiography (right) of the right eye.

**Figure 3:**
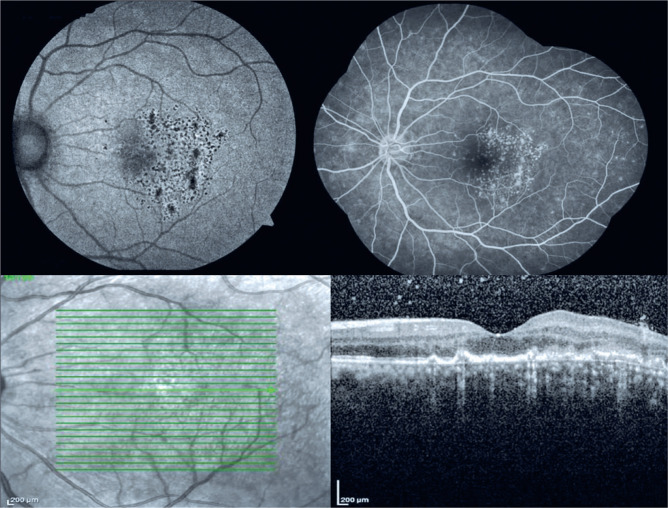
Autofluorescence (top left), fluorescein angiography (top right), and optical coherence tomography (bottom) of the left eye.

**Figure 4:**
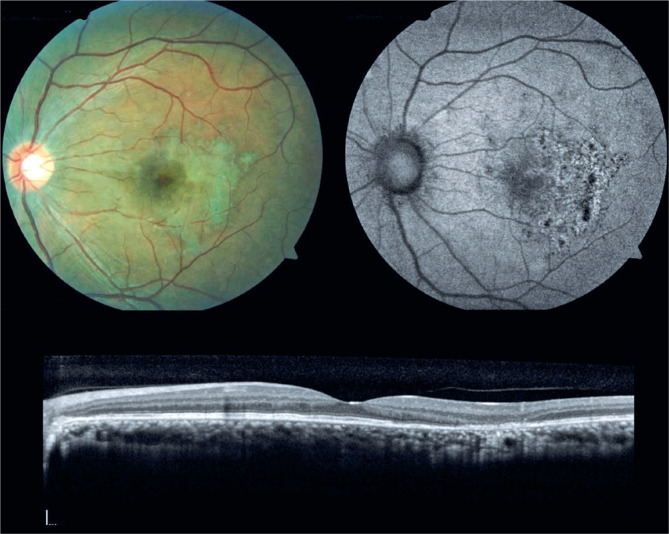
Fundus imaging (top left), autofluorescence (top right), and optical coherence tomography (bottom) of the left eye 30 days after increasing the dose of corticosteroids.

A 32-year-old Caucasian female presented with diminished visual acuity in the left eye (LE) for 3 weeks associated with metamorphopsia and nasal scotoma. Her past medical history included myasthenia gravis (MG), which was being treated for the past 12 years with 150 mg azathioprine, 5 mg prednisone, and 120 mg pyridostigmine. Her family history was unre markable, and epidemiological evaluation for infectious diseases showed negative findings. Her best corrected visual acuity (BCVA) was 20/20 J1 (-0.50/-0.25 × 180°) and 20/100 J5 (-0.25/-0.25 × 170°). Intraocular pressure was 12 mmHg in both eyes, and there were no changes in the anterior segment, except for discrete left ptosis. Fundus examination revealed clear vitreous, isolated, punctate, yellowish perimacular lesions in her right eye and multiple yellow-white lesions that were confluent in the macula and punctate in the periphery in her LE (Figure 1). Laboratory findings, including the venereal disease research laboratory (VDRL) test, the fluorescent treponemal antibody absorption test (FTA-ABS), and hepatitis serology, were negative. Tuberculin skin test reading was 5 mm, although the immunoglobulin release assay (IGRA) showed negative result for *Mycobacterium tuberculosis*. FAF demonstrated hypoautofluorescence with hyperfluorescent margins (Figures 2, 3) corresponding to the lesions observed in both eyes, and FA rev ealed hyperfluorescence since early phases without late leakage (Figures 2, 3). SD-OCT disclosed areas of intermittent RPE elevations, disruption of the ellipsoid zone, and sub-RPE hyporeflectance (Figure 3). The patient was diagnosed with PIC and then treated with an increased prednisone dose of 1 mg/kg/day. After 2 weeks of treatment, her BCVA and imaging status were improved, and within 30 days, she achieved complete visual recovery, with pigmentary alterations remaining on the fundus examination (Figure 4).

## DISCUSSION

PIC is a relatively rare idiopathic inflammatory multifocal chorioretinopathy that affects primarily, but not exclusively, young white myopic women. PIC lesions most commonly involve the posterior pole, not being associated with vitreous or anterior chamber inflammation, which is the primary feature differentiating PIC from MCP. The lesions raise at the level of RPE and inner choroid, sometimes being associated with the development of subretinal fibrosis and choroidal neovascularization (CNV)^(1-4)^. PIC is commonly bilateral, although asymmetrical, with the majority of patients presenting with unilateral symptoms, including scotomata, blurred vision, photopsia, and metamorphopsia^(2)^. Fundoscopic features of PIC comprise small, yellow-white spots that are normally limited to the posterior pole. In our case, these features were somewhat different, with the lesions being found as far as the equator and confluent in the LE, resembling APMPPE, which presents with numerous, creamy-colored, placoid and flat lesions, often forming plaques larger than PIC lesions^(3)^.

Considering that the signs and symptoms tend to overlap in many of the WDS, a multimodal imaging classification was proposed to facilitate their diagnoses and pathological understanding, including, among others, tests such as SD-OCT, FAF, FA, and ICG^(3)^. Based on this classification, we were able to identify, through a multimodal approach, the clinical features that defined the diagnosis of PIC. SD-OCT was of particular importance, which revealed the typical RPE elevations, the ellipsoid layer disruption, and the hyporeflectance underneath, defining it as stage 3 in PIC progression according to Zhang et al.^(5-7)^. In FA, the lesions appeared as punctate hyperfluorescent choroidal lesions in early and late phases, typical of PIC and different from what would be expected of APMPPE lesions, which exhibit hypofluorescence in early phases. In FAF, the lesions were hypoautofluorescent with hyperautofluorescent margins, similar to those in PIC^(3,8)^.

Management of PIC is challenging, with the treatments ranging from observation to intensive immunosuppression or intravitreal anti-VEGF therapy. Some patients have long periods of quiescence, with long-term remission, but some have sight-threatening episodes. Although there is a lack of evidence suggesting a beneficial role for corticosteroids, their use in the acute phase appears to abbreviate the course and lead to stabilization. Immunosuppressive therapy has been used in recurrent cases with sight-threatening lesions, although there is no consensus on which drug has the best profile. CNV lesions, when present, respond well to anti-VEGF therapy, putting aside photodynamic therapy as the first line of treatment, due to its high recurrence rates^(2,4,9,10)^. In our case, the patient was already using the immunosuppresants azathioprine and prednisone. Therefore, after excluding an infectious cause, we established an increase in the corticosteroid intake of up to 1 mg/kg/day, which confirmed to be beneficial for the fast recovery of functional and anatomical aspects.

The visual outcome in PIC depends on the location of lesions and the presence of complications such as subretinal fibrosis and CNV. Spontaneous recovery or successful treatment and absence of complications are associated with good prognosis^(2)^.

The WDS configure a diagnostic challenge for ophthalmologists, primarily considering their possible overlap and similarities. With multimodal imaging, differentiation has become tangible, allowing for a more specific diagnosis and management. It is of paramount importance to exclude infectious, neoplastic, and other inflammatory conditions for adequate treatment and better prognosis. Treatment must be individually addressed considering the extent of the disease, the risk of progression, and the patient’s circumstances.
